# Sarcopenia and Risk of Cognitive Impairment: Cohort Study and Mendelian Randomization Analysis

**DOI:** 10.2196/66031

**Published:** 2025-06-11

**Authors:** Tingting Sha, Yuqing Zhang, Jie Wei, Changjun Li, Chao Zeng, Guanghua Lei, Yilun Wang

**Affiliations:** 1 Department of Orthopaedics, Xiangya Hospital, Central South University, 87 Xiangya Road, Changsha, 410008, China, 86 84327326; 2 Hunan Key Laboratory of Joint Degeneration and Injury, Changsha, China; 3 Key Laboratory of Aging-related Bone and Joint Diseases Prevention and Treatment of Ministry of Education, Changsha, China; 4 Division of Rheumatology, Allergy, and Immunology, Department of Medicine, Massachusetts General Hospital, Harvard Medical School, Boston, MA, United States; 5 The Mongan Institute, Massachusetts General Hospital, Harvard Medical School, Boston, MA, United States; 6 National Clinical Research Center for Geriatric Disorders, Xiangya Hospital, Central South University, Changsha, China; 7 Department of Epidemiology and Health Statistics, Xiangya School of Public Health, Central South University, Changsha, China; 8 Department of Endocrinology, Endocrinology Research Center, Xiangya Hospital of Central South University, Changsha, China

**Keywords:** sarcopenia, musculoskeletal disease, cognitive impairment, cognition, mediation, Mendelian randomization, genetic variation, cohort study

## Abstract

**Background:**

Over half the people over 60 years of age experience cognitive impairment, with limited treatment options, making it crucial to identify risk factors. Studies have examined the association between sarcopenia and cognitive impairment; however, the evidence is inconclusive and cannot be used to make causal inferences.

**Objective:**

This study aims to appraise the causal association of sarcopenia with cognitive impairment by triangulating the data from a cohort study and Mendelian randomization (MR) analysis.

**Methods:**

Using UK Biobank data, we first examined the associations of sarcopenia and its indices (appendicular lean mass [ALM], handgrip strength, and gait speed) with cognitive function (fluid intelligence and prospective memory) by using mixed-effects regression models. Then, we explored the causal associations of genetically predicted sarcopenic indices with cognitive function through a 2-sample MR, and examined potential mediation by omega-3 fatty acids, vitamin D levels, physical inactivity, falls, frailty, sleep disorders, anxiety, depression, stroke, metabolic syndrome, and type 2 diabetes.

**Results:**

A total of 34,457 participants, with a mean age of 56.4 (SD 7.6) years, 51.1% (n=17,620) of which were female, completed baseline cognitive tests between 2006 and 2010 and attended at least 1 follow-up visit in 2012, 2014, or 2019, and were included in the observational analysis. The cohort study revealed that sarcopenia was significantly associated with cognitive impairment, which was evidenced by reduced fluid intelligence scores (β=−0.91, 95% CI −1.68 to −0.15; *P*=.02). Each of the sarcopenic indices also exhibited significant associations with either fluid intelligence or prospective memory (all *P*<.05). MR analyses yielded compelling evidence of positive associations between the genetically predicted increases in ALM (β=0.09, 95% CI 0.07-0.12; *P*<.001), handgrip strength (β=0.18, 95% CI 0.08-0.29; *P*<.001) and gait speed (β=0.78, 95% CI 0.53-0.29; *P*<.001) and improved cognitive function. The effects of ALM and handgrip strength on cognitive function were partially mediated by genetically predicted physical activity, with indirect effects of 0.01 (95% CI 0.00-0.02) for ALM and 0.02 (95% CI 0.00-0.05) for handgrip strength.

**Conclusions:**

Our study suggests that sarcopenia is a potential causal risk factor for cognitive impairment, with physical activity acting as a modifiable mediator in this relationship.

## Introduction

An impairment in cognitive function is a neurodegenerative process that affects 10%‐20% of people aged 65 years and older [1], and that can lead to adverse health consequences, including diminished quality of life [2] and increased risk of hospitalization [3], as well as mortality [4]. However, by the time of diagnosis, the pathological changes related to cognitive impairment have often become irreversible. The available treatments are limited and target symptoms, with very low efficacy [5]. Thus, identifying the potential factors for predicting the risk of subsequent cognitive impairment has become a public health priority [6,7]. Such measurable indices can help recognize high-risk populations to explore potential disease-modifying therapies.

Sarcopenia, characterized by accelerated loss of muscle mass and deterioration in function, is a prevalent skeletal muscle disorder in the elderly [8]. As predicted by statistics, about 2 billion individuals globally will be affected by sarcopenia by 2050 [9]. Through the progression of physical inactivity, patients with sarcopenia tend to experience an increased risk of disability, which has been associated with cognitive impairment due to its impact on neurogenesis and cerebral blood vessel formation [10,11]. Previous cohort studies have reported a possible relationship between sarcopenia and cognitive impairment [12-17]. However, this relationship is still not fully understood and may even be controversial [18]. Some studies have even rejected any significant association between sarcopenia itself [19], or its related indices [20,21] and cognitive impairment. Discrepancies in definitions of sarcopenia and sample sizes among studies may underlie these conflicting outcomes. Hence, a more intricate exploration is warranted to establish the precise link between sarcopenia and cognitive impairment.

Furthermore, current available human evidence on such association is mostly based on observational research, and therefore cannot be relied on to derive causal inferences due to inherent limitations (eg, reverse causality and unmeasured confounders) [22]. Examining the causal association between sarcopenia and cognitive impairment and identifying potentially modifiable intervention targets along the causal pathway is of important public health significance for preventing cognitive impairment [18]. Mendelian randomization (MR) is a method that involves using genetic variants as proxies for the targeted exposure. Since, genetic variants are conditional on parental genotypes and randomly allocated at conception, the results from MR studies are more resistant to reverse causality and confounding than those derived from conventional observational studies [22].

Using a large population-based cohort, this study was initiated by evaluating the observational data that links the consensus definition of sarcopenia, which was established by the European Working Group on Sarcopenia in Older People in 2019 (EWGSOP2), as well as its 3 defining indices (ie, appendicular lean mass [ALM], handgrip strength, and gait speed), to the risk of cognitive impairment. To best assess causality, we performed multiple MR analyses to explore the potential causal association between sarcopenia and cognitive function. Subsequently, we further used MR mediation analysis to examine the degree to which 11 putative mediators (ie, omega-3 fatty acids, vitamin D levels, physical inactivity, falls, frailty, sleep disorders, anxiety, depression, stroke, metabolic syndrome, and type 2 diabetes) may impact the effects of sarcopenia, to identify potential intervention targets in the causal pathway. This study aims to elucidate the causal relationship between sarcopenia and cognitive impairment while identifying actionable intervention targets within the causal pathway through the evaluation of these mediators.

## Methods

### Study Design

We examined the associations of sarcopenia and its indices with cognitive function in a cohort of approximately 35,000 participants from the United Kingdom Biobank. We then conducted multiple MR analyses to explore the potential causal relations between sarcopenic indices (ie, ALM, handgrip strength, and gait speed) and cognitive function. Finally, we used mediation MR analysis to quantify to what extent physical inactivity, depression, anxiety, falls, frailty, and vitamin D use mediated the effects of sarcopenic indices on cognitive function. This research has been conducted using the UK Biobank Resource under Application 77,646.

### Cohort Study

#### Study Participants

The UK Biobank is a comprehensive prospective cohort that enrolled ≥500,000 participants aged 40‐69 from 22 centers over the United Kingdom between 2006 and 2010. Neuropsychological tests were performed to assess cognitive function assessment in a subset of participants at baseline as well as 3 subsequent visits via a touch screen. Approximately 170,000 individuals participated in the baseline visit (2006‐2010), and ~20,000, ~60,000, and ~8000 individuals participated in the first (2012‐2013), second (2014+), and third (2019+) follow-up visits, respectively. For each follow-up visit dataset, consistent diagnostic criteria were applied for both sarcopenia and cognitive function. In our analysis, we included 35,000 participants who underwent cognitive function tests at baseline and had at least one subsequent follow-up visit (see Figure S1 in Multimedia Appendix 1).

#### Exposure and Outcome Measurements

Sarcopenia was defined according to the EWGSOP2, which uses the detection of low grip strength and low muscle mass to confirm sarcopenia, with poor physical performance indicating severe sarcopenia [23]. ALM was evaluated by bioelectrical impedance analysis with a Tanita BC418MA body composition analyzer and expressed in kilograms. We used the cut points as recommended in the EWGSOP2 definition of <7 kg/m^2^ in men and 5.5 kg/m^2^ in women. Grip strength was measured with a hydraulic handheld dynamometer (Jamar J00105). Specifically, 3 measurements were recorded for the maximum strength of both hands, and the highest values were used for analysis, expressed in kilograms. The cut-points for low grip strength recommended by EWGSOP2 were <27 kg in men and <16 kg in women. Gait speed was acquired directly from the participants through the question “How would you characterize your usual walking speed?” (options: “slow” [<3 miles per hour], “steady/average” [3‐4 miles per hour], “fast” [>4 miles per hour], or “prefer not to answer” [regarded as missing data]). Participants who reported that they were unable to walk or walked at a slow pace had low physical performance.

Cognitive function was assessed through 2 neuropsychological tests, namely fluid intelligence and prospective memory, by using a touch screen during visits to the UK Biobank assessment center. The fluid intelligence test was designed as a 13-item problem-solving task aiming at logic and reasoning abilities. Prospective memory, which is a form of episodic memory, was used as an indicator to measure the participants’ capacity to remember future tasks. The participants were instructed to reproduce a figure on a touch screen following a single instruction to be recalled later in the session. The responses were judged as either “correct on the first attempt” or otherwise, indicating a lapse in prospective memory (eg, “instruction not remembered, either skipped or incorrect” or “correctly recalled on the second attempt”).

#### Covariate Measurements

The selection of potential confounders was based on previous literature [12,13], which was assessed through a touch-screen questionnaire at baseline. These variables included: age (continuous), sex (male or female), education level (college or university; A levels (Advanced Level), AS level (Advanced Subsidiary Level) or equivalent; O level, GCSEs (General Certificate of Secondary Education) or equivalent; CSEs or equivalent; NVQ (National Vocational Qualification), HND (Higher National Diploma), HNC (Higher National Certificate) or equivalent; other professional qualifications), self-reported race (White, Asian, Black, Mixed or other), assessment center, Townsend Deprivation Index at recruitment (continuous), BMI (continuous), presence of long-standing illness (no or yes), overall health rating (excellent, good, fair or poor), smoking status (never, former, or current), frequency of alcohol intake (daily or almost daily; 3 or 4 times a week; once or twice a week; 1 to 3 times a month or special occasions only), sleep duration (<7 h/day, 7‐9 h/day, or >9 h/day), and TV viewing duration (continuous). Specifically, the presence of long-standing illness was assessed using the following question, “Do you have any long-standing illness, disability, or infirmity”. Overall health rating was collected by asking “In general how would you rate your overall health?” All categorical variables strictly adhered to UK Biobank classification standards, with no additional category merging or processing.

#### Statistical Analyses

To address the repeated measurements of the 2 cognitive tests, we used mixed-effects linear and logistic regression models to evaluate the associations of sarcopenia and its indices with fluid intelligence scores and with the risk of prospective memory impairment, respectively. All the mixed-effects models featured a random individual-specific intercept with a fixed slope. In regression models, we adjusted for sociodemographic, socioeconomic factors, and the number of cognitive assessments, and then further added health-related factors, including BMI, long-standing illness, and health rating. Finally, lifestyle factors, including smoking, alcohol intake frequency, sleep duration, and duration of TV viewing were added. We used a missing indicator approach in the primary analysis and conducted a sensitivity analysis by excluding missing data to evaluate its impact.

### MR Analyses

#### Data Source of Sarcopenic Indices and General Cognitive Function

Given the lack of genome-wide association studies (GWASs) specifically focused on sarcopenia, we used the publicly available summary data on sarcopenic indices and cognitive function from the UK Biobank [24,25] and the COGENT Consortium [26]. The GWAS summary data for ALM (n=450,243), handgrip strength (n=461,089), and gait speed (n=461,089) were derived from the largest public GWASs of individuals with European ancestry in the UK Biobank [24,25]. The summary genetic statistics for the associations of general cognitive function were extracted from a comprehensive GWAS conducted through the UK Biobank and COGENT Consortium [26]. The summary phenotype source and outcome descriptions are provided in Table S1 in Multimedia Appendix 1.

#### Genetic Instruments for Sarcopenic Indices

The single nucleotide polymorphism (SNPs) meeting the following criteria were selected as instrumental variables: (1) significantly associated with ALM, handgrip strength, or usual gait speed (*P*<5×10^−8^), (2) independent (linkage disequilibrium r^2^<.001 within 10,000 kb), (3) with appropriate effect allele frequencies (≥1%), and (4) not palindromic (adenine/thymine or cytosine/guanine). To correct for multiple comparisons (3 exposures), the Bonferroni method was used. Associations with *P*<.016 (0.05/3) were deemed statistically significant.

#### Data Analyses

##### The 2-Sample MR Analyses

The associations between genetically predicted sarcopenic indices and general cognitive function were examined by multiplicative random-effects inverse variance weighted (IVW) analysis, which can provide the most accurate and unbiased estimates [27]. Furthermore, we performed MR-Egger method, MR-PRESSO method, and RadialMR method to pinpoint potential violations of MR assumptions and assess the robustness of primary results [27,28]. The RadialMR method identifies outliers influencing MR analysis, and the results are reanalyzed after their removal [29]. A consistent estimate across multiple sensitivity analyses indicates strengthened causal evidence. We also assessed the bias and type 1 error rate for sample overlap using an internet-based calculator [30]. In addition, we reanalyzed the data using the MRlap method, which is robust to biases caused by sample overlap, winner’s curse, and weak instruments [31].

##### Mediation MR Analyses

We performed univariable MR to estimate the effects of sarcopenic indices on 11 genetically predicted putative mediators, ie, omega-3 fatty acids, vitamin D levels, moderate-to-vigorous intensity physical activity (MVPA) during leisure time, falls, frailty, sleep disorders, anxiety, depression, stroke, metabolic syndrome, and type 2 diabetes. Mediators showing causal evidence were selected for multivariable Mendelian randomization (MVMR) analysis to estimate the indirect effect of sarcopenic indices on cognitive function mediated by each. The mediation proportion was calculated as the indirect effect divided by the total effect on cognitive function, with standard errors estimated by the delta method [32]. If an inconsistent mediation was observed, where the direct effect opposes the indirect effect, no mediation proportion would be estimated [33].

##### Complementary Analyses

We performed bidirectional MR analysis to partially explore the potential reverse causality. The SNPs from GWAS that were significantly associated with general cognitive function (*P*<5×10^−8^) were selected as instrumental variables (selection criteria remained consistent with the ones mentioned earlier). In addition, we used the MR-Steiger method to examine the directionality of the relationship [34].

All analyses were conducted using the R (R Foundation for Statistical Computing) packages *TwoSampleMR* (version 0.5.6), *MVMR* (version 0.3), *MRPRESSO* (version 1.0), and *MRlap* (version 0.0.3.0) in R (version 4.3.0). A *P* value <.05 was considered significant. IVW estimates were deemed causal if consistent with at least one sensitivity analysis and showed no pleiotropy (Egger intercept *P*>.05). Results were reported as odds ratios (ORs), β coefficients, or proportions with 95% confidence intervals (CIs).

### Ethical Considerations

This study had been granted with the UK Biobank research approval by the North West Centre for Research Ethics Committee (11/NW/0382) and informed consent was obtained from all participants. For GWAS datasets, ethical review and approval can be accessed in the original studies. The data used were anonymized to ensure privacy and confidentiality. No compensation was provided to participants.

## Results

**Figure 1.**
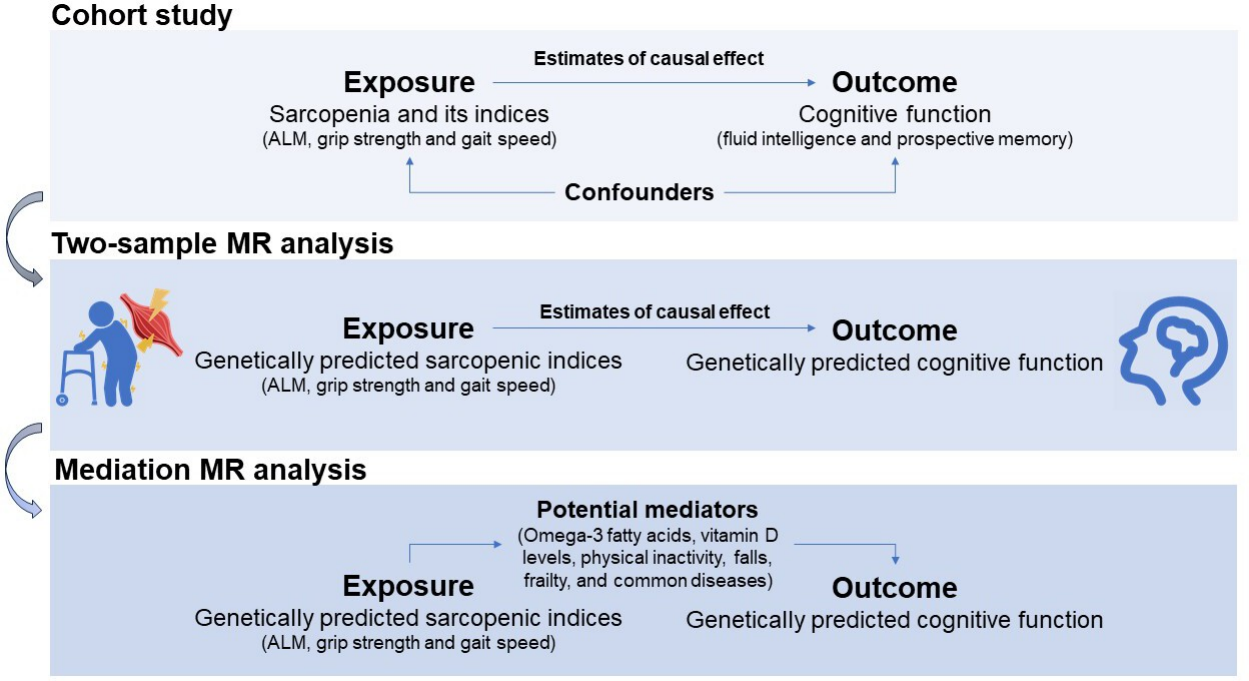
Study design ﬂowchart. ALM: appendicular lean mass; MR: Mendelian randomization.

Figure 1 provides an overview of the study design. The baseline characteristics of the cohort study are summarized in Table 1. Among 34,457 participants, 17,620 (51.1%) were women, with a mean age of 56.4 (SD 7.6) years.

**Table 1.** Baseline characteristics of the cohort study.

Characteristics	Results
Sociodemographics	
Total sample, n	34,457
Age (years), mean (SD)	56.4 (7.6)
Gender (female), n (%)	17,620 (51.1)
Deprivation index, mean (SD)	−1.92 (2.7)
Race, n (%)	
White	33,286 (96.6)
Asian	466 (1.4)
Black	240 (0.7)
Mixed or other	162 (0.5)
Unknown	303 (0.9)
Education level, n (%)	
College or university degree	15,190 (44.1)
A^a^ levels, AS^b^ levels, or equivalent	4355 (12.6)
O levels, GCSEs,^c^ or equivalent	6712 (19.5)
CSEs^d^ or equivalent	1345 (3.9)
NVQ^e^, HND^f^, HNC,^g^ or equivalent	1985 (5.8)
Other professional qualifications	1747 (5.0)
Unknown	3123 (9.1)
Health-related factors	
BMI, n (%)	
Underweight	158 (0.5)
Normal weight	12,792 (37.1)
Overweight	14,684 (46.2)
Obesity	6750 (19.6)
Unknown	73 (0.2)
Long-standing illness, n (%)	
No	24,258 (70.4)
Yes	9571 (27.8)
Unknown	628 (1.8)
Overall health rating, n (%)	
Excellent	7082 (20.6)
Good	20,969 (60.9)
Fair	5541 (16.1)
Poor	796 (2.3)
Unknown	69 (0.2)
Lifestyle behaviors	
Smoking status, n (%)	
Never	20,501 (59.5)
Former	11718 (34.1)
Current	2163 (6.3)
Unknown	75 (0.2)
Alcohol intake frequency, n (%)	
Daily or almost daily	7768 (22.5)
Three or four times a week	9408 (27.3)
Once or twice a week	8694 (25.2)
One to three times a month	3755 (10.9)
Special occasions only	3069 (8.9)
Never	1749 (5.1)
Unknown	14 (0.0)
Sleep duration, n (%)	
Short sleep (<7 h/day)	7504 (21.8)
Normal (7‐9 h/day)	26,465 (76.8)
Long sleep (>9 h/day)	386 (1.1)
Unknown	102 (0.3)
TV viewing (h/days) mean (SD)	1.8 (3.0)

a A: Advanced.

b AS: Advanced Subsidiary.

c GCSE: General Certificate of Secondary Education

d CSE: Certificate of Secondary Education.

e NVQ: National Vocational Qualification.

f HND: Higher National Diploma.

g HNC: Higher National Certificate.

### Cohort Study

As shown in Table 2, participants with sarcopenia had lower fluid intelligence scores than those without sarcopenia, and the multivariable-adjusted difference in the mean level of fluid intelligence scores was 0.91 (95% CI −1.68 to −0.15; *P*=.02). However, no statistically significant association with prospective memory loss was detected (OR 1.36, 95% CI 0.31-5.92; *P*=.68). Each 5-kg increase in ALM was found to be associated with an increased fluid intelligence score (β=0.27, 95% CI 0.21-0.32; *P*<.001) and a decreased risk of prospective memory loss (OR 0.87, 95% CI 0.78-0.97; *P*<.001). Likewise, each 5-kg increase in handgrip strength was positively associated with fluid intelligence (β=0.02, 95% CI 0.01-0.04; *P*<.001) and negatively associated with the prospective memory loss (OR 0.89, 95% CI 0.86-0.93; *P*<.001). Furthermore, slow gait speed was associated with a lower fluid intelligence score (β=−0.10, 95% CI −0.23 to 0.03; *P*=.15) and an increased risk of prospective memory loss (OR 1.65, 95% CI 1.23-2.19; *P*<.001). The results of sensitivity analyses, after excluding cases with missing data, were consistent with the overall analyses (see Table S2 in Multimedia Appendix 1).

**Table 2.** The associations of baseline sarcopenia and its indices with follow-up cognitive function.

Sarcopenia indices	Cognitive function	
	Fluid intelligence, β (95% CI)	Prospective memory loss, OR^a^ (95% CI)
Sarcopenia		
Model 1^b^	−0.87 (−1.62 to −0.12)^c^	1.67 (0.47-1.78)
Model 2^d^	−0.86 (−1.61 to −0.11)^c^	1.59 (0.39-6.50)
Model 3^e^	−0.91 (−1.68 to −0.15)^c^	1.36 (0.31-5.92)
Appendicular lean mass		
Model 1^b^	0.17 (0.13-0.21)^c^	0.87 (0.80-0.94)^c^
Model 2^d^	0.27 (0.22-0.32)^c^	0.82 (0.74-0.91)^c^
Model 3^e^	0.27 (0.21-0.32)^c^	0.87 (0.78-0.97)^c^
Handgrip strength		
Model 1^b^	0.03 (0.02-0.05)^c^	0.93 (0.89-0.96)^c^
Model 2^d^	0.03 (0.01-0.04)^c^	0.91 (0.88-0.94)^c^
Model 3^e^	0.02 (0.01-0.04)^c^	0.89 (0.86-0.93)^c^
Slow gait speed		
Model 1^b^	−0.20 (−0.32 to −0.08)^c^	1.64 (1.27-2.12)^c^
Model 2^d^	−0.14 (−0.27 to −0.01)^c^	1.71 (1.30-2.25)^c^
Model 3^e^	−0.10 (−0.23 to 0.03)	1.65 (1.23-2.19)^c^

a OR: odds ratio.

b Model 1 was adjusted for age, sex, race, aged race, education, deprivation index, and the number of cognitive assessments.

c *P*<.05.

d Model 2 was additionally adjusted for health-related factors including BMI and long-standing illness.

e Model 3 was additionally adjusted for smoking, alcohol intake, sleep duration, and TV viewing.

### MR Analyses

#### The 2-Sample MR Analyses

**Figure 2.**
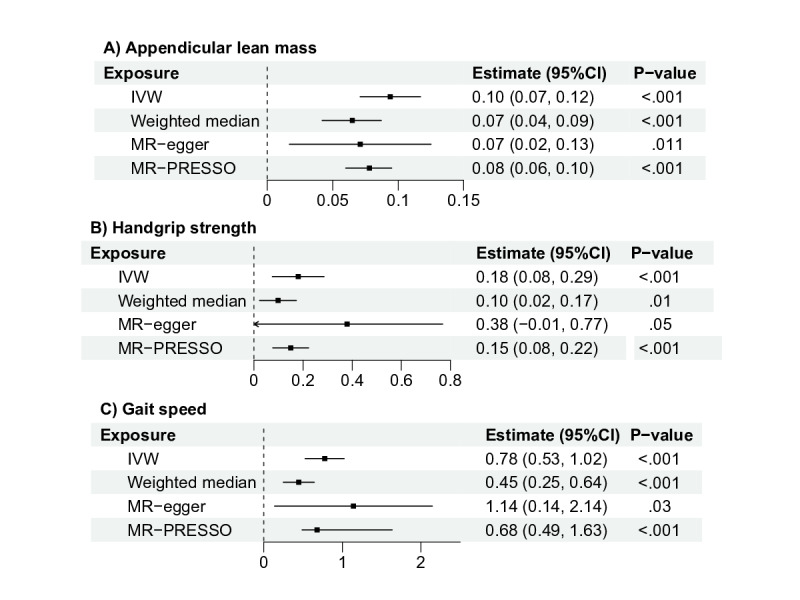
Mendelian randomization results for the relationship of sarcopenic indices with cognitive function. IVW: inverse variance weighted analysis; MR: Mendelian randomization.

As shown in Figure 2, for an increase of each one unit in genetically predicted ALM, handgrip strength, and gait speed, the general cognitive function score was increased by 0.10 (95% CI 0.07-0.12; *P*<.001), 0.18 (95% CI 0.08-0.29; *P*<.001), and 0.78 (95% CI 0.53-1.02; *P*<.001), respectively. To assess the consistency of directional causation, the effect estimates of 3 different sensitivity methods, that is, weighted median, MR-Egger, and MR-PRESSO were examined and plotted, confirming the directional causation between sarcopenic indices to general cognitive function (see Figure 2 and Figure S2 in Multimedia Appendix 1). There was some evidence of heterogeneity in the SNP effects although the MR-Egger intercepts indicated limited evidence of directional pleiotropy (see Table S3 in Multimedia Appendix 1). In radialMR analyses, outliers were detected (see Figure S3 in Multimedia Appendix 1). MR results remained consistent, showing slightly smaller effects after removing outliers (see Table S4 in Multimedia Appendix 1). For all sarcopenic indices and cognitive function phenotypes, the type 1 error rate was robustly controlled below 0.05 and bias estimates were confined to a narrow range of −0.01 to 0.01 (see Table S5 in Multimedia Appendix 1). Validation through the MRlap method further confirmed that sample overlap did not substantially influence the causal inferences (see Table S6 in Multimedia Appendix 1).

#### Mediation MR Analyses

**Figure 3.**
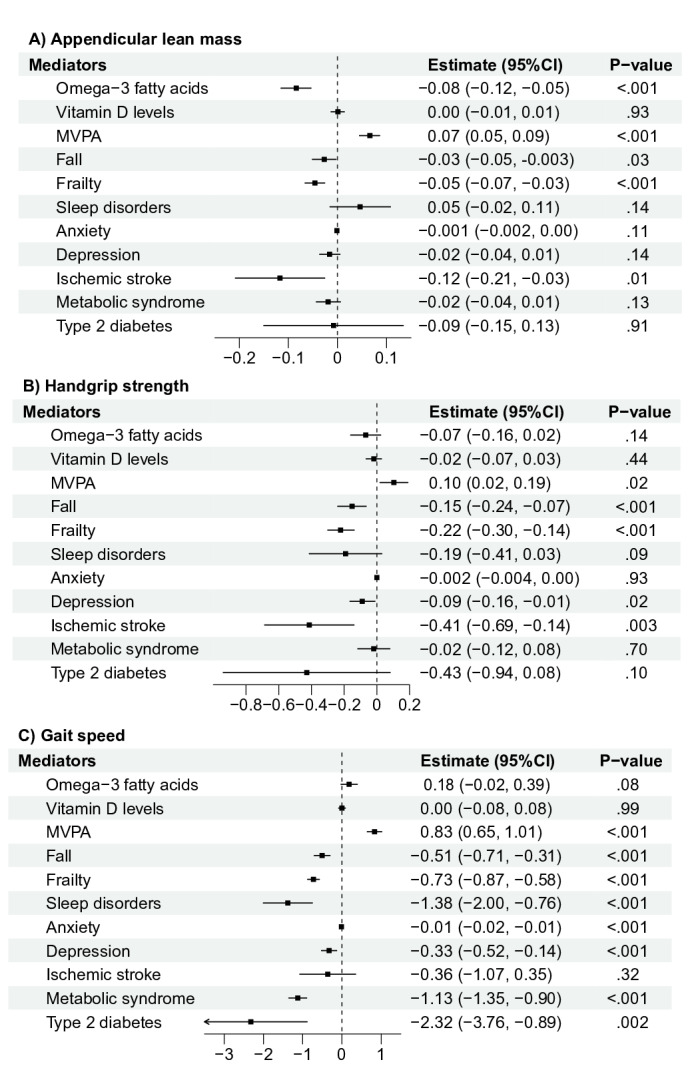
Effects of genetically predicted sarcopenic indices on potential mediators. MVPA: moderate-to-vigorous intensity physical activity.

Figure 3 illustrates the effects of sarcopenic indices on 11 potential mediators. Univariable IVW MR analysis revealed that genetically predicted ALM exhibited protective effects on MVPA (β=0.07, 95% CI 0.05-0.09; *P*<.001) and negative associations with omega-3 fatty acids (β=−0.08, 95% CI −0.12 to −0.05; *P*<.001), falls (β=−0.03, 95% CI −0.05 to -0.003; *P*=.03), and frailty (β=−0.05, 95% CI −0.07 to −0.03; *P*<.001). Genetically predicted handgrip strength showed a positive association with MVPA (β=0.10, 95% CI 0.02-0.19; *P*=.02) and negative associations with falls (β=−0.15, 95% CI −0.24 to −0.07; *P*<.001), and frailty (β=−0.22, 95% CI −0.30 to −0.14; *P*<.001), depression (β=−0.09, 95% CI −0.16 to −0.01; *P*=.02), and stroke (β=−0.41, 95% CI −0.69 to −0.14; *P*=.003). Genetically predicted gait speed was positively associated with MVPA (β=0.83, 95% CI 0.65-1.01; *P*<.001). It also demonstrated significant protective effects against sleep disorders, falls, frailty, anxiety, depression, metabolic syndrome, and type 2 diabetes (with βs ranging from −0.01 to −2.32, all *P* values <.05). Further mediation analysis revealed that the total effect of genetically predicted ALM on general cognitive function decreased from 0.10 (95% CI 0.07-0.12) to 0.09 (95% CI 0.06-0.11) after adjusting for MVPA in MVMR analysis (Table 3). Similarly, the total effect of genetically predicted handgrip strength on general cognitive function attenuated from 0.18 (95% CI 0.08-0.29) to 0.16 (95% CI 0.05-0.27) with adjustment in MVMR analysis. MVPA mediated 8.2% of the total direct effect of ALM on cognitive function, and 10.6% of the total direct effect of handgrip strength on cognitive function. No apparent mediation effect was observed through omega-3 fatty acids, vitamin D level, falls, frailty, sleep disorders, anxiety, depression, stroke, metabolic syndrome, or type 2 diabetes. The type 1 error rate due to sample overlap between sarcopenic indices and the mediators remained below 0.05 (with bias estimates under 0.01) for all phenotypes (see Table S5 in Multimedia Appendix 1).

**Table 3.** The mediation effect of sarcopenic indices on cognitive function via potential mediator in Mendelian randomization (MR) analyses.

Exposures	Mediator	Total effects, β (95% CI)	Direct effects, β (95% CI)	Indirect effects, β (95% CI)	Mediated proportion, % (95% CI)
Appendicular lean mass	MVPA^a^	0.10 (0.07-0.12)^b^	0.09 (0.06-0.11)^b^	0.01 (0.00-0.02)^b^	8.2 (0-16.7)
Handgrip strength	MVPA^a^	0.18 (0.08-0.29)^b^	0.16 (0.05-0.27)^b^	0.02 (0.00-0.05)^b^	10.6 (0-29.6)

a MVPA: moderate-to-vigorous intensity physical activity during leisure time.

b *P*<.05.

#### Complementary Analyses

We conducted bidirectional MR analysis to investigate the potential reverse causality between sarcopenia and cognitive function. Table S7 in Multimedia Appendix 1 shows evidence supporting a causal effect of genetically predicted cognitive function on all sarcopenic indices. The effect estimates demonstrated a general consistency across different sensitivity methods (see Figure S2 in Multimedia Appendix 1). Some heterogeneity in the SNP effects was observed, although the MR-Egger intercepts suggested no evidence of directional pleiotropy (see Table S7 in Multimedia Appendix 1). In radialMR analyses, outliers were detected (see Figure S4 in Multimedia Appendix 1). The MR results remained consistent both before and after outlier correction and are presented in Tables S7 and S8 in Multimedia Appendix 1.

## Discussion

### Principal Findings

This study revealed that sarcopenia and its defining indices (appendicular lean mass, handgrip strength, and gait speed) are associated with cognitive function based on observational data. MR analyses further established a causal relationship between higher levels of sarcopenic indices and better general cognitive function. In addition, physical activity was identified as a significant mediator in the causal pathway linking sarcopenic indices to cognitive function. Our findings suggest sarcopenia as a risk factor and potential biomarker for cognitive impairment, with physical activity offering a therapeutic approach to delay or prevent cognitive decline.

### Comparison With Previous Work

The association between sarcopenia and the risk of cognitive impairment has been investigated by several longitudinal studies, but the results were conflicting. Some authors indicated that individuals with sarcopenia [12-17], reduced muscle strength [13,17,35], decreased muscle mass [35], or compromised physical performance [35,36] were associated with an elevated risk of cognitive impairment. However, some others reported no significant association between sarcopenia or its indicators and the risk of cognitive impairment [19-21]. The conflicting results could stem from differences in how sarcopenia is defined and the variations in sample sizes. Our observational analyses addressed these discrepancies by focusing on the more recent EWGSOP2 definition of sarcopenia and its 3 defining indices within a substantial sample size (>200,000 participants). As a result, we found that sarcopenia, as a construct, and its 3 distinct indices were closely correlated with cognitive impairment. Furthermore, the discrepancies observed in previous observational studies might also be attributed to factors such as residual confounding and measurement errors. To mitigate these limitations in our study, we used MR, a genetic epidemiological technique using genetic variants as proxies for the exposure [37]. This approach is less vulnerable to the aforementioned limitations since the genetic variants are accurately measured and documented and are randomly allocated during gamete formation and conception. Therefore, this method can minimize the potential for measurement errors and reduce the likelihood of being influenced by confounding variables [37].

### Possible Explanations

Our study demonstrates that physical inactivity is a potential mediating factor in the causal pathway between sarcopenia and cognitive impairment. On one hand, individuals with sarcopenia might have a low level of physical activity [38], which could be attributed to the fact that weakened muscles might hinder the ability to exercise regularly. On the other hand, physical activity positively impacts brain health [39]. First, it can stimulate the formation of new neurons, enhance neuronal survival [10], increase resistance to brain injuries, and facilitate synaptic development and plasticity [40]. Second, physical activity promotes better blood vessel formation in the brain, which is associated with increased learning capabilities [10,11]. Third, it also activates specific gene expression profiles benefiting brain plasticity and cognitive function [41]. Fourth, engaging in physical activity has been associated with reduced amyloid deposition in the brains of cognitively normal elderly adults [42]. Furthermore, physical activity can reduce systemic inflammatory markers [43] and boost the production of neuroprotective proteins like brain-derived neurotrophic factor (BDNF), supporting the growth and survival of neurons [44]. In addition, it positively influences energy balance and glucose metabolism by modulating AMP kinase and insulin signaling, potentially aiding Aβ clearance [45]. These multiple benefits of physical activity contribute to safeguarding cognitive function and underscore the importance of maintaining an active lifestyle in individuals with sarcopenia to support brain health.

### Limitations

The triangulation of findings through complementary cohort and MR approaches significantly bolstered the confidence in our drawn inferences. However, several limitations warrant consideration. First, despite using multiple MR methods to resist pleiotropy-related confounding, we could not eliminate residual confounding, which is a known limitation of the MR approach. Second, due to a mere 5% response rate and healthy volunteer bias in the UK Biobank, whether our findings can be generalized to the broader UK population remains uncertain, despite the large sample size. Third, although we have accounted for common modifiable lifestyle factors and preventable diseases to inform public health policies, this study does not encompass all potential mediation pathways. Fourth, only self-reported gait speed was collected, lacking objective measurements for more accurate estimates. Fifth, the inability to perform stratified analyses by age and gender due to data constraints limits insights into these factors’ roles in the sarcopenia-cognition relationship. Sixth, the overlap between GWAS datasets in the MR analysis could bias results and inflate Type 1 error rates. To address this, we assessed the error rate and applied the MRlap method, confirming the robustness of our findings and minimizing the influence of sample overlap on causal associations. Finally, to maintain homogeneity, we focused on individuals of European ancestry in our sample selection, which might influence the generalization of the results, although the sample size was substantial.

### Clinical and Research Implications

Our findings provide a promising approach to alleviate the burden of cognitive impairment by identifying and intervening in sarcopenia. Specifically, we have recognized sarcopenia as a risk factor for future cognitive impairment, making it a potential clinical biomarker to screen adults at risk of late-life cognitive impairment. Although no pharmaceutical treatment has been specifically approved for sarcopenia, implementing non-pharmacological interventions, such as physical activity, can serve as a therapeutic approach to proactively delay or prevent the onset of cognitive impairment in affected individuals. Our research indicates that physical activity can mediate the effect of sarcopenia on cognitive function, offering valuable insights that complement the prevailing emphasis on intellectual pursuits as the primary means of exercising the brain [46]. Promoting physical activity may yield a dual positive impact, addressing both sarcopenia and cognitive impairment simultaneously. This approach can potentially enhance the overall health and well-being of those affected by sarcopenia.

### Conclusions

Our study suggests that sarcopenia is a causal risk factor for cognitive impairment. Physical activity, a modifiable factor, is capable of measuring the effect of sarcopenia on cognitive function.

## Supplementary material

10.2196/66031 Multimedia Appendix 1 Supplementary methods and results for the study.

## Abbreviations

A level: Advanced Level
ALM: appendicular lean mass
AS level: Advanced Subsidiary Level
EWGSOP2: European Working Group on Sarcopenia in Older People in 2019
GCSE: General Certificate of Secondary Education
GWAS: genome-wide association study
HNC: Higher National Certificate
HND: Higher National Diploma
IVW: inverse variance weighted analysis
MR: Mendelian randomization
MVMR: multivariable Mendelian randomization
MVPA: moderate-to-vigorous intensity physical activity
NVQ: National Vocational Qualification
OR: odds ratio
SNP: single nucleotide polymorphism
