# Supplementary material for: Sarcopenia and Risk of Cognitive Impairment: Cohort Study and Mendelian Randomization Analysis
Source: JMIR Aging. 2025 Jun 11;8:e66031. doi: 10.2196/66031 (PMC12176244; doi:10.2196/66031)
Supplement: Multimedia Appendix 1 [file aging-v8-e66031-s001.docx]

**Web Appendix**

**Sarcopenia and risk of cognitive impairment: a cohort study and Mendelian randomization analysis**

**Table of Contents**

[**Supplementary Methods** 2](#_Toc192711113)

[**Supplementary Table S1. GWAS summary statistics: source and description** 4](#_Toc192711114)

[**Supplementary Table S2. The associations of baseline sarcopenia and its indices with follow-up cognitive function after excluding cases with missing data** 5](#_Toc192711115)

[**Supplementary Table S3. Mendelian randomization results for the relationship of sarcopenic indices with cognitive function** 6](#_Toc192711116)

[**Supplementary Table S4. Mendelian randomization results for the relationship of sarcopenic indices with cognitive function after excluding outliers** 7](#_Toc192711117)

[**Supplementary Table S5. Type 1 error rate for the causal estimates due to sample overlap (for exposure and outcome datasets with partial sample overlap)** 8](#_Toc192711118)

[**Supplementary Table S6. MRlap estimates for the causal associations between sarcopenic indices, and cognitive function** 10](#_Toc192711119)

[**Supplementary Table S7. Mendelian randomization results for the relationship between cognitive function and sarcopenic indices** 11](#_Toc192711120)

[**Supplementary Table S8. Mendelian randomization results for the relationship between cognitive function and sarcopenic indices after excluding outliers** 12](#_Toc192711121)

[**Supplementary Figure S1. Diagram of participants in the observational analyses.** 13](#_Toc192711122)

[**Supplementary Figure S2. Mendelian randomization scatter plot of sarcopenia indices with cognitive function (right panels), cognitive function and sarcopenia indices (left panels).** 14](#_Toc192711123)

[**Supplementary Figure S3. Radial plots of sarcopenic indices with cognitive function.** 15](#_Toc192711124)

[**Supplementary Figure S4. Radial plots of cognitive function with sarcopenic indices.** 16](#_Toc192711125)

**Supplementary Methods**

**Data analyses**

***Two-sample MR analyses***

The causal associations between sarcopenic indices and general cognitive function were examined by two-sample MR based on the summary-level genetic data obtained from public GWASs. The associations between genetically predicted sarcopenic indices and general cognitive function were examined by multiplicative random-effects inverse variance weighted (IVW) analysis. The change in cognitive function (βs) and its 95% confidence intervals (95%CIs) per one-unit increase in genetically predicted sarcopenic indices were then calculated for comparative analysis. Scatter plots were utilized to visualize the results. Provided that all SNPs are valid instrumental variables or that the horizontal pleiotropy is balanced, this approach can provide the most accurate and unbiased estimates [1]. To evaluate the robustness of our findings, we examined Cochran’s Q statistic, which assesses heterogeneity among individual genetic variants. This analysis helps identify potential issues with instrument validity. Furthermore, we performed several MR sensitivity analyses by applying the weighted median method, MR-Egger method, MR-PRESSO method, and RadialMR method to pinpoint potential violations of MR assumptions and assess the robustness of primary results. It was found that the weighted median method yielded consistent estimates when ≥50% of the weights originated from valid instrumental variables. The MR-Egger method, while capable of detecting and correcting directional pleiotropy, was limited by a low power [1]. The MR-PRESSO method could help identify and eliminate outlier variants to rectify the potential horizontal pleiotropy and the detected heterogeneity [2]. The RadialMR method with modified second-order weights could help identify outliers that exert significant influence in MR analysis and contribute substantially to Cochran’s Q statistic for heterogeneity [3]. Once an outlier was identified, the results would be re-analyzed after removing it. A consistent estimate across multiple sensitivity analyses indicates strengthened causal evidence. We also assessed the bias and type 1 error rate for sample overlap using an online calculator (<https://sb452.shinyapps.io/overlap/>) [4]. Additionally, we reanalyzed the data using the MRlap method, which is robust to biases caused by sample overlap, winner’s curse, and weak instruments [5].

**References**

1. Burgess S, Bowaen J, Fall T, Ingelsson E, Thompson SG. Sensitivity Analyses for Robust Causal Inference from Menaelian Ranaomization Analyses with Multiple Genetic Variants. Epiaemiology. 2017 Jan;28(1):30-42. PMIA: 27749700. aoi: 10.1097/EAE.0000000000000559.

2. Verbanck M, Chen CY, Neale B, Ao R. Aetection of wiaespreaa horizontal pleiotropy in causal relationships inferrea from Menaelian ranaomization between complex traits ana aiseases. Nat Genet. 2018 May;50(5):693-8. PMIA: 29686387. aoi: 10.1038/s41588-018-0099-7.

3. Bowaen J, Spiller W, Ael Greco MF, Sheehan N, Thompson J, Minelli C, et al. Improving the visualization, interpretation ana analysis of two-sample summary aata Menaelian ranaomization via the Raaial plot ana Raaial regression. Int J Epiaemiol. 2018 Aug 1;47(4):1264-78. PMIA: 29961852. aoi: 10.1093/ije/ayy101.

4. Burgess S, Aavies NM, Thompson SG. Bias aue to participant overlap in two-sample Menaelian ranaomization. Genet Epiaemiol. 2016 Nov;40(7):597-608. PMIA: 27625185. aoi: 10.1002/gepi.21998.

5. Mounier N, Kutalik Z. Bias correction for inverse variance weighting Menaelian ranaomization. Genet Epiaemiol. 2023 Jun;47(4):314-31. PMIA: 37036286. aoi: 10.1002/gepi.22522.

**Supplementary Table S1. GWAS summary statistics: source and description**

| **Phenotypes** | **Sample size** | **Year** | **PubMed ID** | **GWAS ID** |
| --- | --- | --- | --- | --- |
| Appendicular lean mass | 450,243 | 2020 | 33097823 | ebi-a-GCST90000025 |
| Handgrip strength | 459,915 | 2018 | 29846171 | ukb-b-10215 |
| Gait speed | 459,915 | 2018 | 29846171 | ukb-b-4711 |
| Cognitive function | 257,841 | 2018 | 30038396 | ebi-a-GCST006572 |
| Omega-3 fatty acids | 114,999 | 2020 | - | met-d-Omega_3 |
| Vitamin D level | 79,366 | 2018 | 29343764 | ebi-a-GCST005367 |
| Moderate-to-vigorous intensity physical activity during leisure time | 606,820 | 2022 | 36071172 | - |
| Falls | 451,179 | 2020 | 32999390 | ebi-a-GCST90012857 |
| Frailty | 175,226 | 2021 | 34431594 | ebi-a-GCST90020053 |
| Anxiety | 462,933 | 2018 | 29846171 | ukb-b-17243 |
| Depression | 180,866 | 2016 | 27089181 | ebi-a-GCST003769 |
| Sleep disorders | 216,700 | 2021 | - | finn-b-SLEEP |
| Metabolic syndrome | 291,107 | 2019 | 31589552 | - |
| Ischemic stroke | 29,633 | 2016 | 26935894 | ieu-a-1108 |
| Type 2 diabetes | 298,957 | 2018 | 29632382 | ebi-a-GCST007515 |

**Supplementary Table S2. The associations of baseline sarcopenia and its indices with follow-up cognitive function after excluding cases with missing data**

| **Sarcopenia indices** | **Cognitive function** | |
| --- | --- | --- |
|  | **Fluid intelligence**  **β (95%CI)** | **Prospective memory loss**  **OR (95%CI)** |
| Sarcopenia |  |  |
| Model 1 | -0.884 (-1.593, -0.174) ^*^ | 1.63 (0.43, 6.25) |
| Model 2 | -0.869 (-1.580, -0.158) ^*^ | 0.59 (0.15, 2.27) |
| Model 3 | -0.869 (-1.590, -0.148) ^*^ | 1.52 (0.35, 6.52) |
| Appendicular lean mass index | |  |
| Model 1 | 0.171 (0.130, 0.211) ^*^ | 0.85 (0.79, 0.92) ^*^ |
| Model 2 | 0.274 (0.220, 0.327) ^*^ | 0.88 (0.80, 0.97) ^*^ |
| Model 3 | 0.272 (0.218, 0.326) ^*^ | 0.84 (0.75, 0.95) ^*^ |
| Handgrip strength |  |  |
| Model 1 | 0.032 (0.015, 0.049) ^*^ | 0.89 (0.86, 0.92) ^*^ |
| Model 2 | 0.025 (0.007, 0.042) ^*^ | 0.92 (0.88, 0.95) ^*^ |
| Model 3 | 0.021 (0.003, 0.038) ^*^ | 0.93 (0.90, 0.96) ^*^ |
| Slow gait speed |  |  |
| Model 1 | -0.187 (-0.302, -0.071) ^*^ | 1.96 (1.51, 2.54) ^*^ |
| Model 2 | -0.120 (-0.238, -0.001) ^*^ | 1.54 (1.16, 2.03) ^*^ |
| Model 3 | -0.079 (-0.197, 0.039) | 1.47 (1.11, 1.94) ^*^ |

CI, confidence interval; OR, odds ratio.

Model 1 was adjusted for age, sex, race, age* race, education, deprivation index, and the number of cognitive assessments. Model 2 was additionally adjusted for health-related factors including body mass index, and long-standing illness. Model 3 was additionally adjusted for smoking, alcohol intake, sleep duration, and TV viewing.

*P<0.05.

**Supplementary Table S3. Mendelian randomization results for the relationship of sarcopenic indices with cognitive function**

| **Exposure** | **MR method** | **Estimates (95% CI)** | ***P*** | ***P* for Cochran’s Q** | ***P* for Egger intercept** |
| --- | --- | --- | --- | --- | --- |
| Appendicular lean mass | IVW | 0.094 (0.071, 0.117) | 1.10×10^-15^ | <0.001 |  |
|  | Weighted median | 0.065 (0.042, 0.087) | 1.27×10^-8^ |  |  |
|  | MR Egger | 0.071 (0.017, 0.125) | 0.011 | <0.001 | 0.350 |
|  | MR-PRESSO | 0.078 (0.060, 0.095) | 8.21×10^-16^ |  |  |
| Handgrip strength | IVW | 0.180 (0.075, 0.285) | 7.42×10^-4^ | <0.001 |  |
|  | Weighted median | 0.098 (0.023, 0.172) | 0.010 |  |  |
|  | MR Egger | 0.379 (-0.009, 0.768) | 0.057 | <0.001 | 0.497 |
|  | MR-PRESSO | 0.150 (0.077, 0.222) | 8.68×10^-5^ |  |  |
| Gait speed | IVW | 0.775 (0.528, 1.022) | 7.62×10^-10^ | <0.001 |  |
|  | Weighted median | 0.446 (0.250, 0.641) | 7.95×10^-6^ |  |  |
|  | MR Egger | 1.141 (0.138, 2.144) | 0.030 | <0.001 | 0.435 |
|  | MR-PRESSO | 0.678 (0.488, 1.633) | 1.51×10^-10^ |  |  |

MR, Mendelian randomization; CI, confidence interval; IVW, inverse variance weighted method.

**Supplementary Table S4. Mendelian randomization results for the relationship of sarcopenic indices with cognitive function after excluding outliers**

| **Exposure** | **MR method** | **Beta (95% CI)** | ***P*** | ***P* for Cochran’s Q** | ***P* for Egger intercept** |
| --- | --- | --- | --- | --- | --- |
| Appendicular lean mass | IVW | 0.076 (0.062, 0.090) | 4.22×10^-26^ | 0.661 |  |
|  | Weighted median | 0.065 (0.041, 0.088) | 4.45×10^-8^ |  |  |
|  | MR Egger | 0.061 (0.028, 0.094) | 3.34×10^-4^ | 0.661 | 0.350 |
|  | MR-PRESSO | 0.076 (0.062, 0.090) | 4.22×10^-26^ |  |  |
| Handgrip strength | IVW | 0.169 (0.114, 0.225) | 2.07×10^-9^ | 0.134 |  |
|  | Weighted median | 0.145 (0.066, 0.224) | 3.28×10^-4^ |  |  |
|  | MR Egger | 0.348 (0.144, 0.553) | 1.15×10^-3^ | 0.169 | 0.118 |
|  | MR-PRESSO | 0.169 (0.114, 0.225) | 2.35×10^-8^ |  |  |
| Gait speed | IVW | 0.715 (0.563, 0.866) | 2.48×10^-20^ | 0.167 |  |
|  | Weighted median | 0.693 (0.486, 0.900) | 5.61×10^-11^ |  |  |
|  | MR Egger | 0.756 (0.148, 1.363) | 2.16×10^-2^ | 0.138 | 0.815 |
|  | MR-PRESSO | 0.715 (0.563, 0.866) | 5.36×10^-10^ |  |  |

MR, Mendelian randomization; CI, confidence interval; IVW, inverse variance weighted method.

**Supplementary Table S5. Type 1 error rate for the causal estimates due to sample overlap (for exposure and outcome datasets with partial sample overlap)**

| **Phenotype 1** | **Phenotype 2** | **Sample size of Phenotype 1** | **Sample size of Phenotype 2** | **Sample overlap proportion** | **No. of SNP as IV** | **R^2^** | **Observational estimate** | **Bias** | **Type 1 error rate** |
| --- | --- | --- | --- | --- | --- | --- | --- | --- | --- |
| Sample overlap between each exposure and outcome | | | |  |  |  |  |  |  |
| Appendicular lean mass | Cognitive function | 450,243 | 257,841 | 0.49 | 565 | 0.119 | 0.158 | 0.001 | 0.05 |
| Handgrip strength | Cognitive function | 459,915 | 257,841 | 0.48 | 170 | 0.016 | 0.064 | 0.001 | 0.05 |
| Gait speed | Cognitive function | 459,915 | 257,841 | 0.48 | 58 | 0.005 | 0.115 | 0.001 | 0.05 |
| Sample overlap between each exposure and mediators | | | |  |  |  |  |  |  |
| Appendicular lean mass | MVPA | 450,243 | 606,820 | 0.73 | 565 | 0.119 | -0.118 | 0.001 | 0.05 |
| Appendicular lean mass | Depression | 450,243 | 180,866 | 0.02 | 565 | 0.119 | 0.194 | 0.000 | 0.05 |
| Appendicular lean mass | Anxiety | 450,243 | 462,933 | 0.97 | 565 | 0.119 | 0.009 | 0.000 | 0.05 |
| Appendicular lean mass | Falls | 450,243 | 451,179 | 0.99 | 565 | 0.119 | 0.209 | -0.002 | 0.05 |
| Appendicular lean mass | Frailty | 450,243 | 175,226 | 0.36 | 565 | 0.119 | 0.020 | 0.000 | 0.05 |
| Handgrip strength | MVPA | 459,915 | 606,820 | 0.73 | 170 | 0.016 | 0.242 | 0.003 | 0.05 |
| Handgrip strength | Depression | 459,915 | 180,866 | 0.02 | 170 | 0.016 | -0.199 | 0.000 | 0.05 |
| Handgrip strength | Anxiety | 459,915 | 462,933 | 0.97 | 170 | 0.016 | -0.153 | -0.003 | 0.05 |
| Handgrip strength | Falls | 459,915 | 451,179 | 0.99 | 170 | 0.016 | -0.257 | -0.006 | 0.05 |
| Handgrip strength | Frailty | 459,915 | 175,226 | 0.36 | 170 | 0.016 | -0.064 | -0.001 | 0.05 |
| Gait speed | MVPA | 459,915 | 606,820 | 0.73 | 58 | 0.005 | 0.373 | 0.005 | 0.05 |
| Gait speed | Depression | 459,915 | 180,866 | 0.02 | 58 | 0.005 | -0.336 | -0.001 | 0.05 |
| Gait speed | Anxiety | 459,915 | -0.1500 | 0.97 | 58 | 0.005 | -0.150 | 0.000 | 0.05 |
| Gait speed | Falls | 459,915 | 451,179 | 0.99 | 58 | 0.005 | -0.279 | -0.001 | 0.05 |
| Gait speed | Frailty | 459,915 | 175,226 | 0.36 | 58 | 0.005 | -0.062 | 0.000 | 0.05 |

Since the observational estimates were not available from previous studies, we applied the estimates in our study as the substitute parameter.

**Supplementary Table S6. MRlap estimates for the causal associations between sarcopenic indices, and cognitive function**

| **Exposure** | **Outcome** | **Observed β** | **Observed 95% CI** | **Observed P** | **Corrected β** | **Corrected 95% CI** | **Corrected P** | **Test difference** | **P for difference** |
| --- | --- | --- | --- | --- | --- | --- | --- | --- | --- |
| Appendicular lean mass | Cognitive function | 0.084 | 0.069, 0.099 | <0.001 | 0.083 | 0.068, 0.099 | <0.001 | 0.23 | 0.815 |
| Handgrip strength | Cognitive function | 0.144 | 0.084, 0.204 | <0.001 | 0.159 | 0.088, 0.230 | 0.001 | -1.37 | 0.170 |
| Gait speed | Cognitive function | 0.450 | 0.304, 0.595 | <0.001 | 0.574 | 0.376, 0.772 | <0.001 | -4.89 | <0.001 |

**Supplementary Table S7. Mendelian randomization results for the relationship between cognitive function and sarcopenic indices**

| **Outcome** | **MR method** | **Estimates (95% CI)** * | ***P*** | ***P* for Cochran’s Q** | ***P* for Egger intercept** |
| --- | --- | --- | --- | --- | --- |
| Appendicular lean mass | IVW | 0.151 (0.090, 0.213) | 1.41×10^-6^ | <0.001 |  |
|  | Weighted median | 0.080 (0.046, 0.114) | 4.70×10^-6^ |  |  |
|  | MR Egger | 0.174 (-0.096, 0.444) | 0.208 | <0.001 | 0.594 |
|  | MR-PRESSO | 0.101 (0.068, 0.134) | 2.36×10^-8^ |  |  |
| Handgrip strength | IVW | 0.041 (0.008, 0.075) | 0.014 | <0.001 |  |
|  | Weighted median | 0.036 (0.011, 0.061) | 0.005 |  |  |
|  | MR Egger | 0.130 (-0.014, 0.275) | 0.080 | <0.001 | 0.093 |
|  | MR-PRESSO | 0.029 (0.006, 0.052) | 0.014 |  |  |
| Gait speed | IVW | 0.101 (0.077, 0.125) | 2.10×10^-16^ | <0.001 |  |
|  | Weighted median | 0.082 (0.062, 0.103) | 1.52×10^-14^ |  |  |
|  | MR Egger | 0.174 (0.070, 0.280) | 1.39×10^-3^ | <0.001 | 0.125 |
|  | MR-PRESSO | 0.101 (0.077, 0.125) | 9.77×10^-18^ |  |  |

MR, Mendelian randomization; CI, confidence interval; IVW, inverse variance weighted method.

**Supplementary Table S8. Mendelian randomization results for the relationship between cognitive function and sarcopenic indices after excluding outliers**

| **Outcome** | **MR method** | **Beta (95% CI)** * | ***P*** | ***P* for Cochran’s Q** | ***P* for Egger intercept** |
| --- | --- | --- | --- | --- | --- |
| Appendicular lean mass | IVW | 0.130 (0.104, 0.156) | 5.20×10^-23^ | 0.125 |  |
|  | Weighted median | 0.111 (0.074, 0.149) | 4.93×10^-9^ |  |  |
|  | MR Egger | 0.135 (0.03, 0.241) | 1.44×10^-2^ | 0.110 | 0.849 |
|  | MR-PRESSO | 0.130 (0.104, 0.156) | 3.24×10^-15^ |  |  |
| Handgrip strength | IVW | 0.027 (0.010, 0.044) | 0.002 | 0.739 |  |
|  | Weighted median | 0.037 (0.012, 0.061) | 0.003 |  |  |
|  | MR Egger | 0.041 (-0.036, 0.118) | 0.297 | 0.716 | 0.714 |
|  | MR-PRESSO | 0.027 (0.005, 0.048) | 0.021 |  |  |
| Gait speed | IVW | 0.087 (0.072, 0.101) | 9.44×10^-34^ | 0.373 |  |
|  | Weighted median | 0.076 (0.056, 0.096) | 1.76×10^-13^ |  |  |
|  | MR Egger | 0.042 (-0.022, 0.108) | 0.204 | 0.396 | 0.230 |
|  | MR-PRESSO | 0.087 (0.073, 0.101) | 4.13×10^-21^ |  |  |

MR, Mendelian randomization; CI, confidence interval; IVW, inverse variance weighted method.


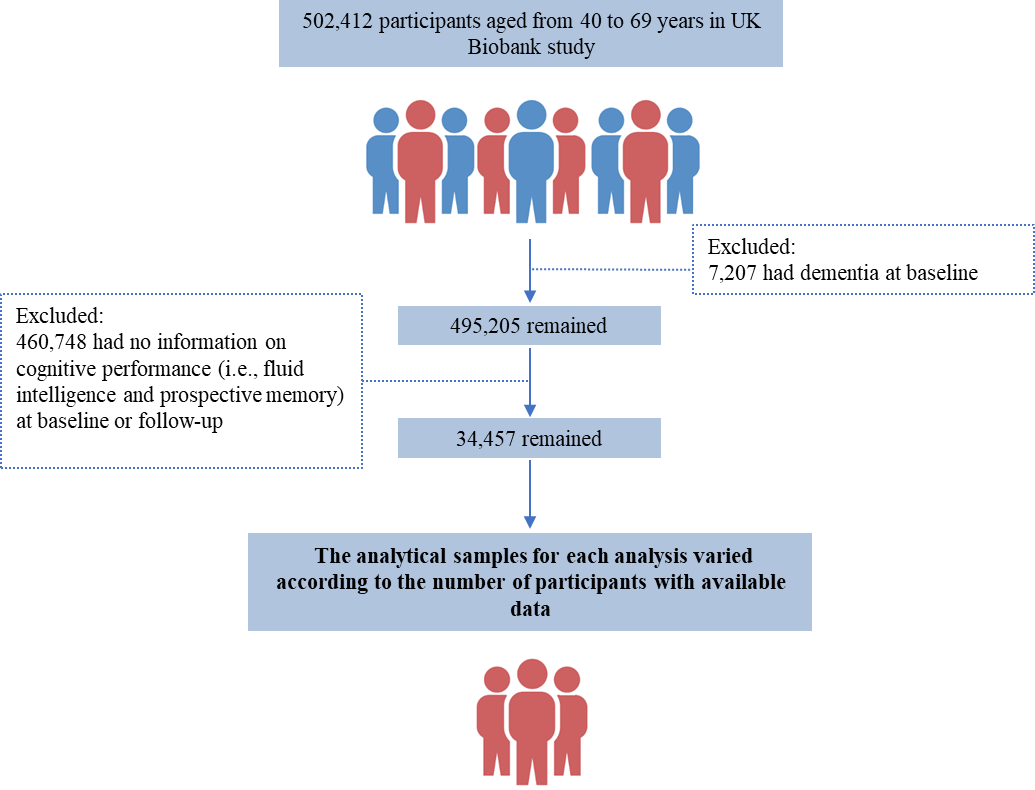


**Supplementary Figure S1. Diagram of participants in the observational analyses.**


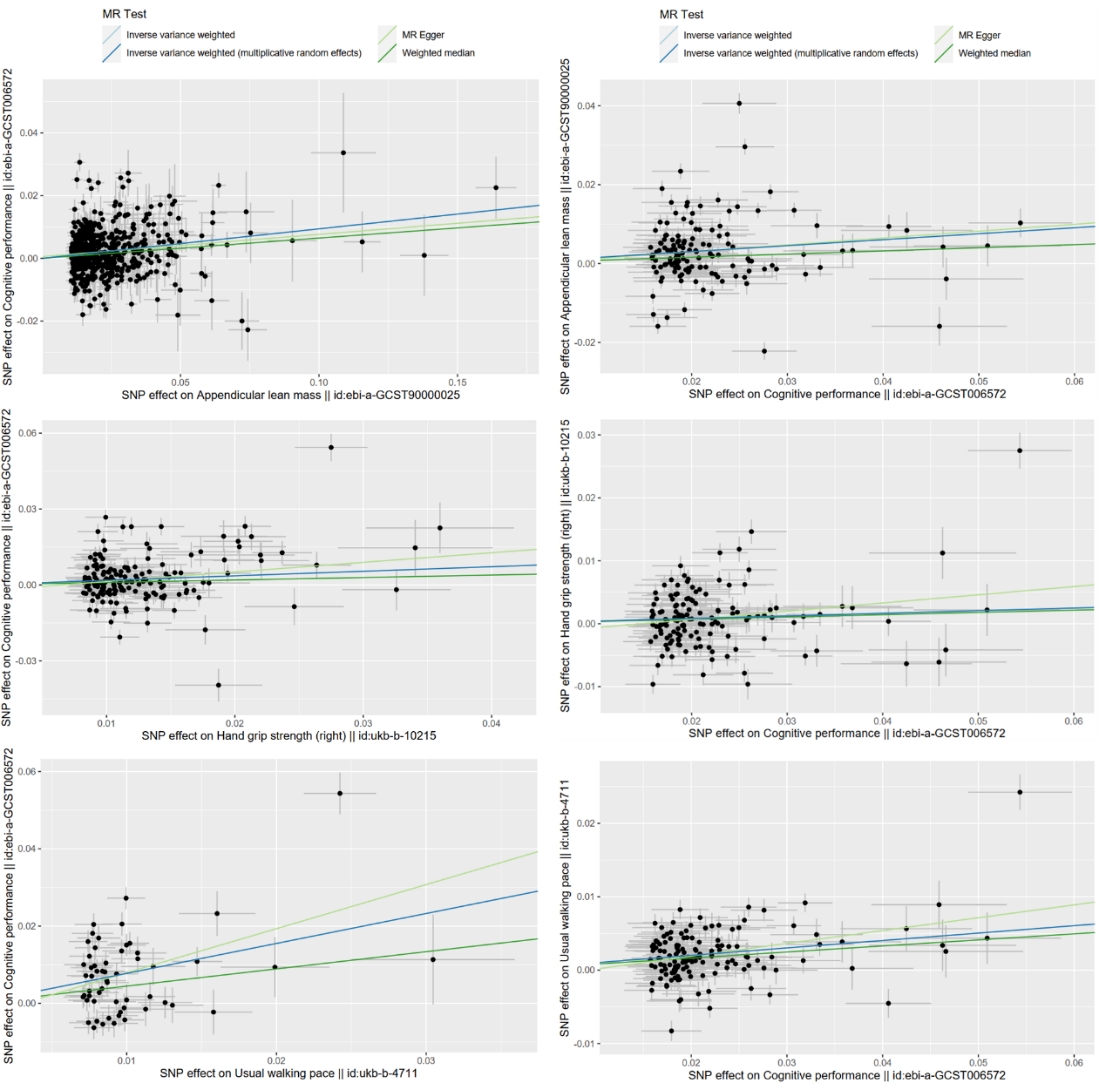


**Supplementary Figure S2. Mendelian randomization scatter plot of sarcopenia indices with cognitive function (right panels), cognitive function, and sarcopenia indices (left panels).**


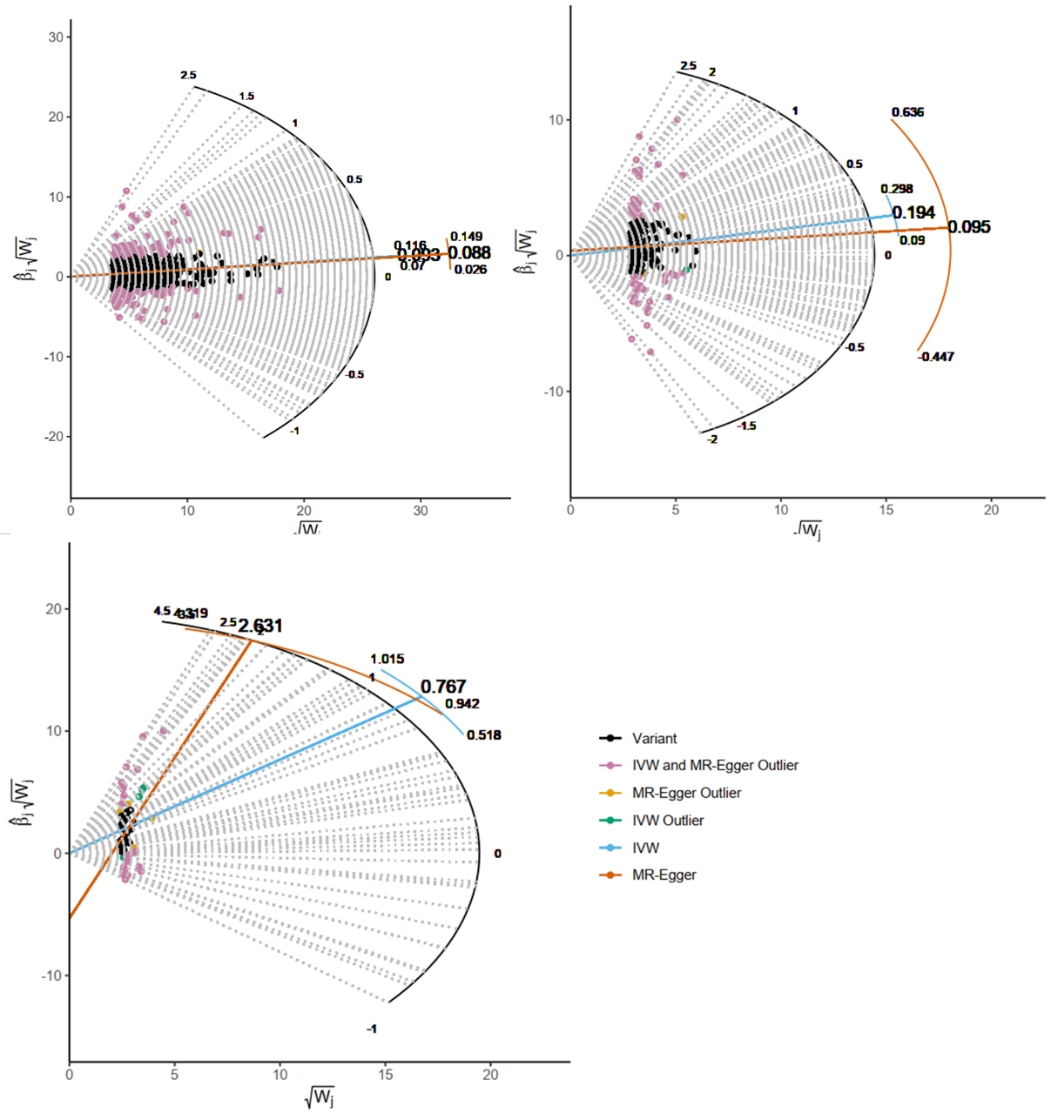


**Supplementary Figure S3. Radial plots of sarcopenic indices with cognitive function.**


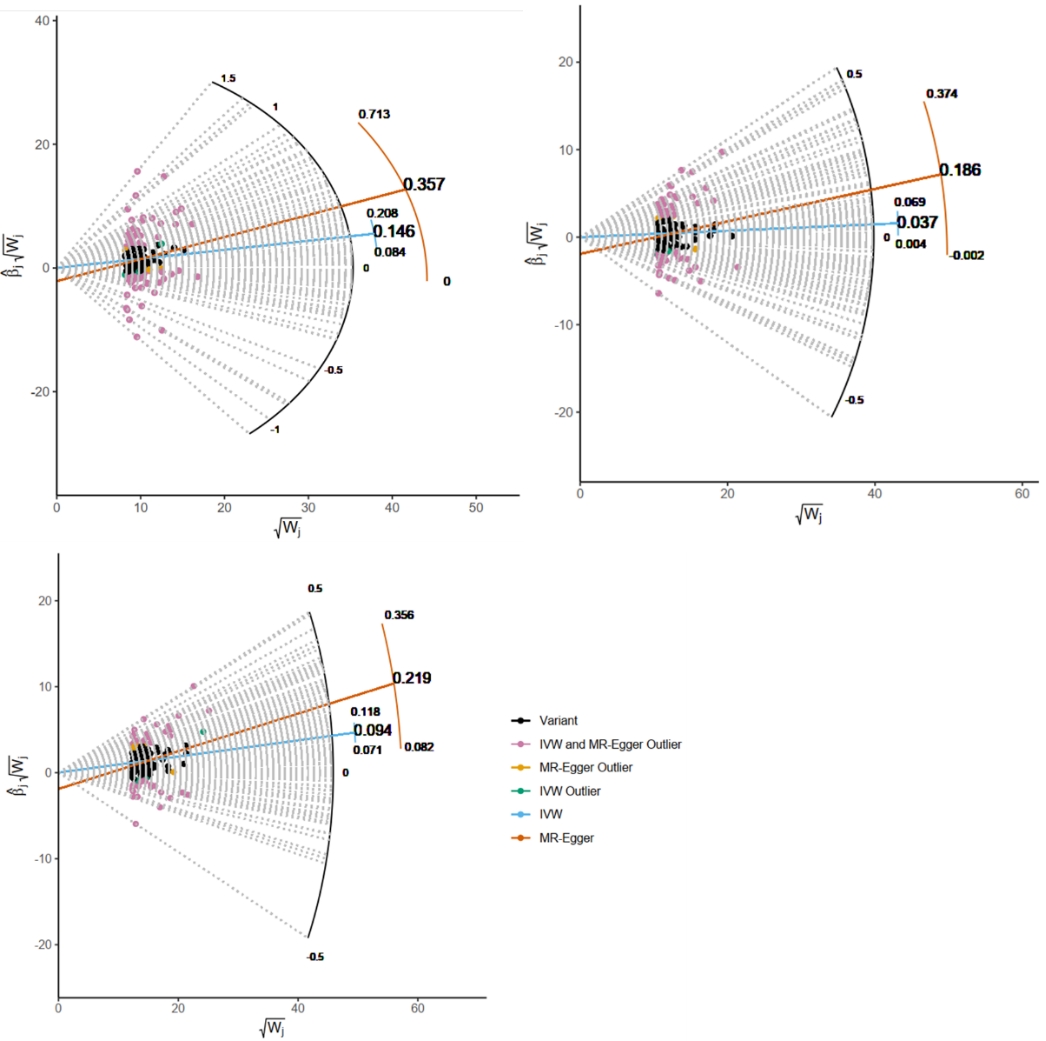


**Supplementary Figure S4. Radial plots of cognitive function with sarcopenic indices.**
